# Promising Biomarkers of Environmental Enteric Dysfunction: A Prospective Cohort study in Pakistani Children

**DOI:** 10.1038/s41598-018-21319-8

**Published:** 2018-02-14

**Authors:** Najeeha Talat Iqbal, Kamran Sadiq, Sana Syed, Tauseefullah Akhund, Fayyaz Umrani, Sheraz Ahmed, Mohammad Yawar Yakoob, Najeeb Rahman, Shahida Qureshi, Wenjun Xin, Jennie Z. Ma, Molly Hughes, Syed Asad Ali

**Affiliations:** 1 Department of Pediatrics and Child Health, Aga Khan University, Karachi, Pakistan; 2 Department of Biological and Biomedical Sciences, Aga Khan University, Karachi, Pakistan; 3 Department of Pediatrics, University of Virginia, Charlottesville, VA USA; 4 Department of Public Health Sciences, University of Virginia, Charlottesville, VA USA; 5 Department of Medicine, University of Virginia, Charlottesville, VA USA

## Abstract

Environmental Enteric Dysfunction (EED), a syndrome characterized by chronic gut inflammation, contributes towards stunting and poor response to enteric vaccines in children in developing countries. In this study, we evaluated major putative biomarkers of EED using growth faltering as its clinical proxy. Newborns (n = 380) were enrolled and followed till 18 months with monthly anthropometry. Biomarkers associated with gut and systemic inflammation were assessed at 6 and 9 months. Linear mixed effects model was used to determine the associations of these biomarkers with growth faltering between birth and 18 months. Fecal myeloperoxidase (neutrophil activation marker) at 6 months [β = −0.207, p = 0.005], and serum GLP 2 (enterocyte proliferation marker) at 6 and 9 months [6M: β = −0.271, p = 0.035; 9M: β = −0.267, p = 0.045] were associated with decreasing LAZ score. Ferritin at 6 and 9 months was associated with decreasing LAZ score [6M: β = −0.882, p < 0.0001; 9M: β = −0.714, p < 0.0001] and so was CRP [β = −0.451, p = 0.039] and AGP [β = −0.443, p = 0.012] at 9 months. Both gut specific and systemic biomarkers correlated negatively with IGF-1, but only weakly correlated, if at all with each other. We therefore conclude that EED may be contributing directly towards growth faltering, and this pathway is not entirely through the pathway of systemic inflammation.

## Introduction

Environmental Enteric Dysfunction (EED) is a structural and functional disorder of gut that primarily affects children living in resource-poor countries with limited access to standard quality food, water and sanitation^1^. A few studies have highlighted the typical histological features of EED in small bowel biopsies, which include villous blunting, increase in intraepithelial lymphocytes (IELs), crypt hyperplasia and abundance of CD25+ cells^2–4^. Functionally, EED has been hypothesized as a major driver of poor linear and cognitive development in children in developing countries^5,6^. Using poor linear growth rate during the first 18 months of life as a proxy of EED, we aimed to validate putative biomarkers of growth faltering, selected based on their role in gut inflammation [fecal myeloperoxidase (MPO) and neopterin (NEO)], enterocyte regeneration (Reg-1b) and proliferation [Glucagon Like Peptide-2 (GLP2)]. We also measured the association of a few systemic inflammatory biomarkers [serum Ferritin, c-reactive protein (CRP), α1 acid glycoprotein (AGP)] as well as Insulin-Like Growth factor 1 (IGF-1) with linear growth. If validated, these biomarkers could be considered as a monitoring tool in future studies that focus on preventing and treating EED.

## Material and Methods

### Description of cohort and study site

**Figure 1:**
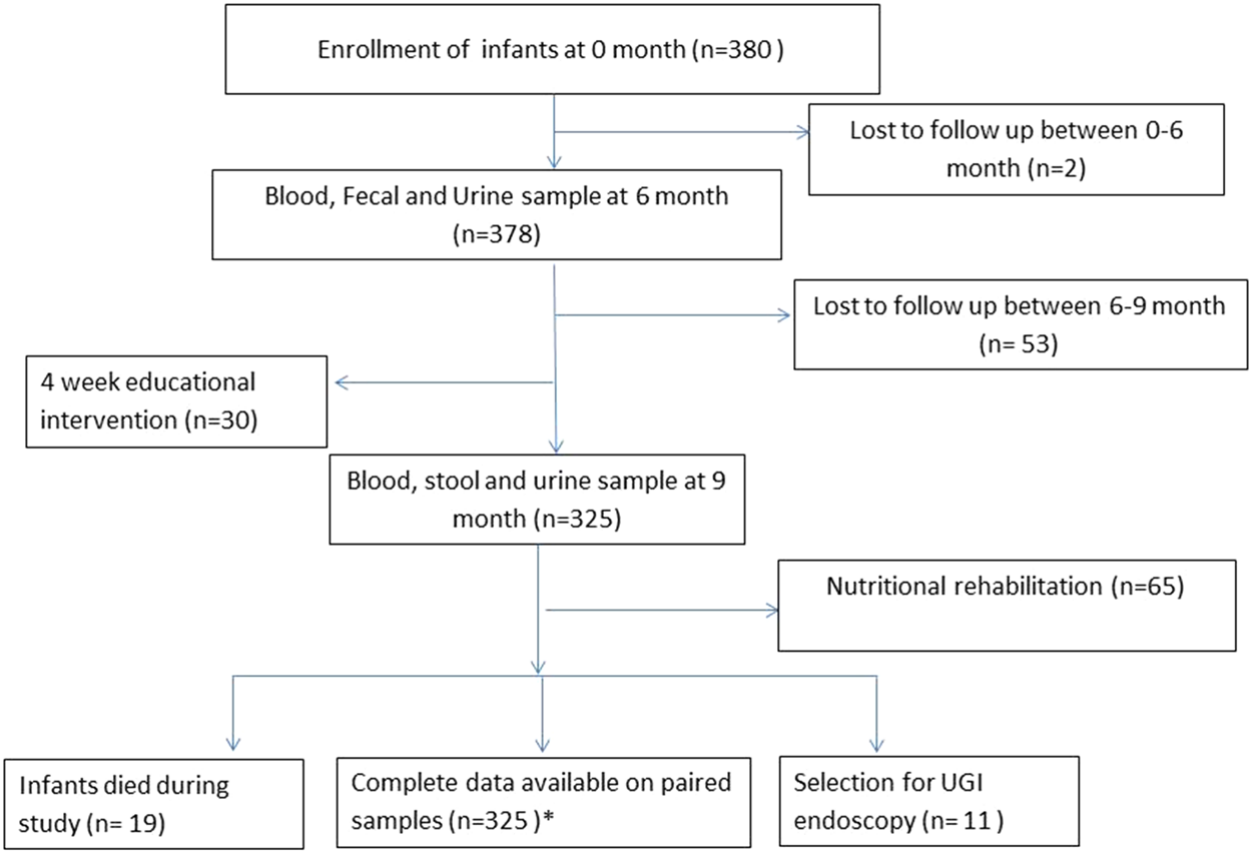
Enrollments of children in the cohort. All newborn were recruited in the cohort through active surveillance within 24 hrs. of birth. Biological samples were collected from children at 6 and 9 month. Monthly anthropometry measurement from birth to 18 months, weekly record of acute respiratory infections (ARI) and diarrhea were recorded through home visits. Between 6 and 9 months nutritional intervention was done on 65 children selected on the basis of WHZ < −2.0. Furthermore 4 week educational session was carried out by showing video to mother of children (http://www.dailymotion.com/video/x1mztre_infant-dietary-counseling-pakistan_lifestyle). Children who showed no improvement in growth after interventions were further evaluated for detailed histopathalogical analysis by two independent physicians. Complete data were obtained on 325 samples where both 6 and 9 months paired samples were available. Of these, *272 had a complete set of all biomarkers analyzed.

The study was a prospective community-based active surveillance of newborns enrolled at birth and followed until 18 months of age. The study was conducted from April 2013 to November 2015 in the rural district of Matiari, located in Sindh province of Pakistan. The Department of Pediatrics and Child Health at Aga Khan University has established research infrastructure in Matiari for community based research^7^. This study was conducted in the selected union councils (administrative divisions), Shah Alam Shah Ji Wasi and Sekhat/Khyber, that had a population of 60,674 and 12,085 children less than 59 months, respectively. The available health facilities in the district include a basic health unit, a rural health center, and a linked referral hospital. Newborns were assessed and enrolled during routine pregnancy surveillance of women of reproductive age (13–49 years) by community health workers. Inclusion criteria for study enrollment included newborns up to 14 days without any major congenital abnormality and whose parents or guardians were willing to provide informed consent. Children were excluded if their families planned to move out of the study area during the next 6 months after enrollment. Enrolled babies were followed till 18 months of age with weekly home visits. During the weekly visits, the community health workers recorded the number of days with reported fever, symptoms of acute respiratory illness, vomiting, and diarrhea in the preceding week. All families were provided a mobile telephone number of the study physicians, in case of any urgent medical need. Monthly anthropometric measurements including height (1 mm precision using a rigid length board with a movable foot piece) and weight (20 g precision electronic scale; TANITA 1584) of all enrolled children were recorded. Standardization of measurements was ensured through regular staff training and cross checks. The anthropometric analysis was done by standard WHO Software (ENA smart 2011, Emergency Nutrition Assessment). Children who were identified as having moderate to severe acute malnutrition (weight for height Z score (WHZ) < −2) at 9 months of age were selected for nutritional and educational rehabilitative interventions. Nutritional intervention included provision of weight appropriate quantity of Ready to Use Therapeutic Food (RUTF) for two months, with close monitoring of compliance. RUTF is a part of nutritional rehabilitation as per World Health Organization’s Integrated Management of Childhood Illnesses guidelines. This nutrition intervention is considered as a home based treatment for children with Severe Acute Malnutrition (SAM). Educational intervention included interactive sessions with the parents and caregivers using standardized training videos about age appropriate complimentary feeding over a month. Compliance to these was monitored during the weekly visits. Figure 1 and Supplementary Fig. S1 show the study flow chart of infants enrolled at birth and details of specimen collected at 6 and 9 months.

### Biological sample collection

Blood urine and fecal specimens were collected from the enrolled subjects at six and nine months of age. 1–2 ml of blood was collected in a gel top tube (BD vaccutainer) by venipuncture, and processed for serum separation within two hours of collection in the field site laboratory located at Matiari. Serum was aliquoted in small volumes as per each assay requirement to prevent repeat freeze thawing. For fecal sample collection, caretaker of a child was instructed to collect fecal sample using a wooden spatula into a clean container provided in stool collection kit. The fecal sample was transferred into multiple cryogenic vials for long term storage. For urine collection, paediatric urine bag was supplied along with diapers. Urine bag was placed by sticking the bag in front of external genitalia, such that it prevents fecal contamination. All biological samples were transported at 4 °C from Matiari field site lab to Infectious Disease Research Lab (IDRL) under cold chain maintenance. All samples were stored at −80 °C until processing for specific assays.

### Measurement of biomarkers

We used commercial ELISA kits for the estimation of GLP-2 (USCN, Life sciences Inc, Wuhan, China) and Reg1B (TechLab, Blacksburg, Virginia). The Reg1B kits were kindly provided by the Petri Lab at University of Virginia for estimation of blood and fecal Reg1B. For intestinal inflammation, we used commercial ELISA kits for the estimation of MPO (Immunodiagnostic AG, Stubenwald-Allee, and Bensheim) and NEO (GenWay Biotech, San Diego. CA) in fecal samples as tested previously in MALED cohort^8^. CRP, ferritin and AGP were analyzed on Hitachi 902 analyzer (Roche Diagnostics, Holliston, MA) and IGF-1 was measured on LIAISON, (Diasorin Saluggia (VC) Italy). All protocols were followed as per manufacturers’ instructions. The final dilution of serum and fecal biomarkers was determined by selecting the most appropriate concentration of a biomarker falling in the linear range of standard curve. Reg1B was performed in two dilutions of 1:40,000 and 1:100,000 for fecal samples, NEO at the dilution of 1:250 and MPO at 1:500. All plates were read on Biorad iMark (Hercules, CA) plate reader.

### Statistical analysis

Two statistical analysis approaches were used to assess the association of biomarkers with linear growth faltering. Firstly, the monthly length for age Z score (LAZ) from birth to 18 months was modeled as the longitudinal responses in a linear mixed effects model. Random intercept and random slope were specified in the model to allow subject-specific variation in LAZ growth. An individual biomarker was considered as a fixed effect, and its association with LAZ was characterized by its interaction with time. That is, the biomarker effect on LAZ was quantified by its influence on the LAZ slope, the rate of LAZ change per year. Due to their skewed distributions, biomarkers were divided into four quartiles as q1 (0–25^th^ percentile), q2 (25^th^–50^th^ percentile), q3 (50^th^–75^th^ percentile) and q4 (75^th^–100^th^ percentile) at 6 and 9 months. The lowest quartile (q1) was used as the reference group. The linear trends in biomarkers with the growth response were evaluated separately in a linear mixed effects model, in which a continuous variable with the median value in each biomarker quartile was considered as a fixed effect, and p-value for its interaction with time was referred to as P-trend. Data was adjusted for covariates such as sex, pre-term birth, maternal age and maternal education status and use of antibiotics. All P trend values are corrected for Type I error using FDR for multiple comparison. This calculation was performed separately for 6- and 9-month biomarkers. Given that these biomarkers may be potentially correlated, such adjustment is necessary to address the risk of false positive discovery (Supplementary Table S1). Secondly, to calculate sensitivity and specificity of biomarkers in predicting poor linear growth, the outcome of linear growth was divided into the following two categories: 1) poor linear growth was defined as change in LAZ < −1 standard deviation (SD) between birth and 18 months; 2) good linear growth was defined as change in LAZ ≥ 0 from birth to 18 months.

Factor analysis was carried out using Varimax rotation on continuous data of biomarker measurements to characterize signature biomarkers of growth faltering. PCA classified biomarkers into main principal components separately for 6 and 9 month measurements. This analysis created a new coordinate system, with the first principal component or PC1 explained the greatest variance, and PC2 and PC3 for the second and third variance in the data. The optimal number of factors were determined by Catell Scree plots using a cutoff of 1 for the sorted Eigenvalues. All p values are 2-sided with p < 0.05 considered as statistically significant. The statistical analyses were performed in SAS, version 9.4 (SAS Institute Inc., Cary, NC).

### Ethics statement

The study protocol was approved by the Ethical Review Committee (ERC) of Aga Khan University in 2013 (ERC: 2446 Ped ERC13). The children were enrolled in the study after written informed consent by parents or legal guardian on behalf of children. All experiments on Human Subject Research were performed in accordance with relevant guidelines and regulations.

## Results

### Baseline characteristics of cohort

Baseline characteristics of children are shown in Table 1. Thirty five percent of children had a weight of less than 2.5 kg at enrollment. Fifty seven percent received colostrum at birth while 14% of children received cow milk at birth.

**Table 1:** Baseline characteristics of children at the time of enrollment.

Characteristics	n	Median (IQR)	Mean (SD)	n (%)
Age at Enrollment (days)	380	4.0 (2–8)	5.58 (4.05)	
Gender M:F	380			
Male				195 (51.3)
Female				185 (48.7)
LAZ at Enrollment	380	−1.37 (−2.23 to −0.47)	−1.39 (1.48)	
WHZ at Enrollment	299	−0.31 (−1.23 to 0.51)	−0.45 (1.45)	
WAZ at Enrollment	380	−1.28 (−2.03 to −0.56)	−1.29 (1.14)	
MUAC at the time of enrollment (cm)	380	8.8 (8.02−9.3)	8.73 (0.87)	
Head Circumference at enrollment (cm)	380	33 (32–34)	32.96 (1.66)	
Antenatal visits	380			
Yes				323 (85.0)
No				57 (15.0)
Gestational Age		36.0 (36–37)	36.59 (1.20)	
Gestational Age (weeks) > = 37	134			(35.3)
Gestational Age (weeks) <37	246			(64.7)
Delivery (home/facility)	380			
Home				124 (32.6)
Maternity home				254 (66.8)
Weight at enrollment				
<2.5 kg	134			(35.3)
≥2.5 kg	245			(64.5)
Colostrum given at birth	380			218 (57.3)
Cow milk given at birth	380			55 (14.5)
BCG vaccine at birth	380			
Yes				81 (21.3)
No				299 (78.7)
OPV vaccine at birth	380			
Yes				89 (23.4)
No				291 (76.6)

**Figure 2:**
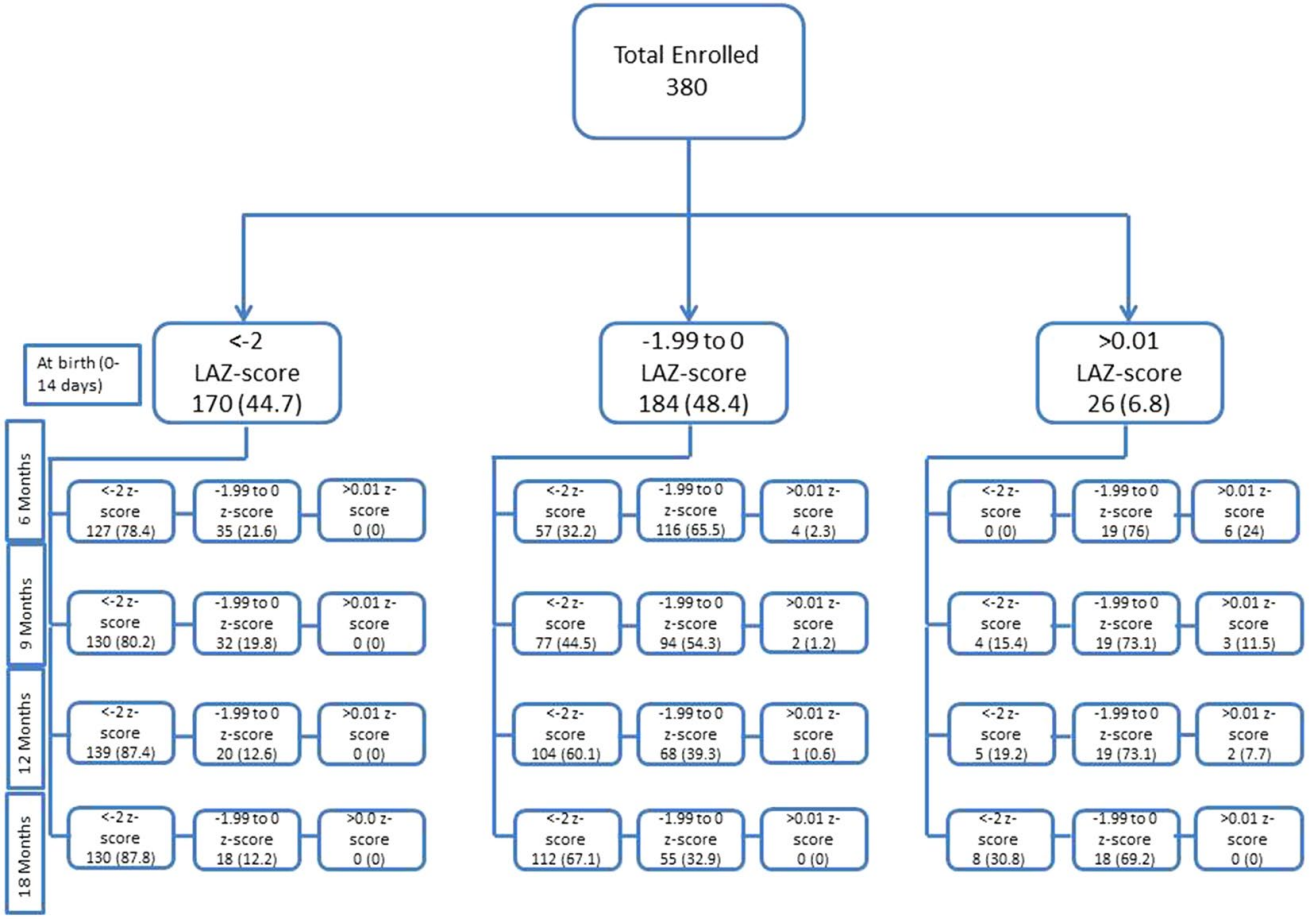
Longitudinal assessment of LAZ scores of children from birth to 18^th^ month. Figure 2 illustrates the longitudinal Z scores of 380 children from birth to 18^th^ month in 3 categories of LAZ (LAZ < −2.0, LAZ between −1.99 to 0 and LAZ > 0.01).

LAZ score trajectory from birth to 18 months on 380 enrolled children is shown in Fig. 2. Overall, most children who were stunted at birth remained stunted for the duration of the study, while children who were between −2 and 0 LAZ upon enrollment had a progressive decline, such that most of them ended up as being LAZ < −2 at 18 months of age. None of the children who had LAZ > 0 upon enrollment maintained their growth trajectory, with most of them ending up with LAZ between 0 and −2 by the time they were 18 months of age.

### Association of EE biomarkers with growth faltering from birth to 18 months

Using linear mixed effects models, we first analyzed the associations of gut specific biomarkers with longitudinal LAZ score (Table 2). All biomarkers were divided in 4 quartiles as q1–q4. The highest quartile (q4) of serum GLP-2 (>429763 ng/ml) was associated with change in LAZ of −0.271 per year (p trend = 0.035) compared to the reference quartile (q1) (<2539 ng/ml) at 6 month, and 0.267 per year (p trend = 0.045) at 9 months when q4 (>75442 ng/ml) was compared with q1 (<3789 ng/ml). Similar analysis for fecal MPO and NEO was carried out at 6 and 9 months. Both biomarkers were tested previously in MALED^8^ and PROVIDE study cohorts as biomarker of intestinal inflammation^9^. Fecal MPO was significantly associated with overall decline in LAZ from birth to 18^th^ months (p trend = 0.005) when tested at 6 month. At 9 month, the significant decline in LAZ of children at the rate of −0.397 and −0.257 and per year was observed, when third (16100 ng/ml) and fourth (80200 ng/ml) quartiles of MPO were compared with the reference quartile (1500 ng/ml) (p = 0.001 and p = 0.042). No association was observed between MPO measured at 9 months and the slope of LAZ. For Neopterin, the linear trend for LAZ decline was marginal but not statistically significant at 6 and 9 month (p = 0.078; p = 0.054).

**Table 2:** Association of biomarkers of Intestinal inflammation, proliferation and regeneration with growth faltering during the first 18 months of life (n = 272).

Biomarker		6 month			9 month		
		*Quartiles cutoff	**Change in HAZ per year compared to q1	(P-trend) p value	Quartiles cutoff	Change in HAZ per year compared to q1	(P-trend) p value
**GLP2 (serum)** (ng/ml)	q1	2539	Ref	**(0.035)**	3789	Ref	**(0.045)**
	q2	7383	0.019	0.880	8513	0.101	0.436
	q3	11817	−0.212	0.099	12346	−0.000	0.998
	q4	429763	−0.271	**0.039**	75442	−0.267	**0.038**
**Fecal Reg-1b** (pg/ml)	q1	73	Ref	(0.694)	79	Ref	(0.646)
	q2	153	−0.084	0.507	178	0.199	0.118
	q3	272	0.031	0.806	328	0.013	0.914
	q4	1237	−0.051	0.688	1377	−0.071	0.576
**Fecal NEO** (nmol/L)	q1	275	Ref	(0.078)	400	Ref	(0.054)
	q2	475	−0.031	0.802	574	0.231	0.063
	q3	725	−0.137	0.268	725	0.239	0.065
	q4	4400	−0.216	0.092	4400	0.013	0.915
**Fecal MPO** (ng/ml)	q1	6050	Ref	**(0.005)**	1500	Ref	(0.302)
	q2	14300	−0.008	0.948	5550	−0.367	**0.003**
	q3	35200	−0.126	0.317	16100	−0.397	**0.001**
	q4	95600	−0.207	0.099	80200	−0.257	**0.042**

We next assessed the systemic inflammatory biomarkers with yearly change in the longitudinal LAZ scores. Higher ferritin levels at both 6 and 9 months were associated with greater decline in LAZ scores (p trend = p < 0.0001). Similarly increase in both AGP and CRP at 9 months of age were associated with yearly decline in LAZ score with a significant P trends for AGP (p = 0.012) and CRP (p = 0.04) (Table 3).

**Table 3:** Association of biomarkers of systemic inflammation with growth faltering during the first 18 months of life. (n = 272).

Biomarker		6 month			9 month		
		*Quartiles cutoff	**Change in HAZ per year compared to q1	(P-trend) p value	Quartiles cutoff	Change in HAZ per year compared to q1	(P-trend) p value
**Ferritin** (ng/ml)	q1	13	Ref	**(<0.001)**	7	Ref	**(<0.001)**
	q2	28	−0.341	**0.003**	14	−0.227	**0.059**
	q3	55	−0.565	<**0.001**	30	−0.501	<**0.001**
	q4	684	−0.882	<**0.001**	370	−0.714	<**0.001**
**CRP** (mg/L)	q1	0.3	Ref	(0.078)	0.3	Ref	**(0.039)**
	q2	1.0	−0.149	0.216	1.4	−0.234	0.062
	q3	3.4	−0.478	<**0.001**	5.5	−0.226	0.072
	q4	58	−0.299	**0.014**	103.3	−0.451	<**0.001**
**AGP** (mg/dl)	q1	72.3	Ref	(0.068)	84.6	Ref	**(0.012)**
	q2	92.4	−0.153	0.244	107.8	−0.234	**0.072**
	q3	118.3	−0.147	0.263	139.8	−0.209	0.106
	q4	343.2	−0.283	**0.032**	312.5	−0.443	<**0.001**
**IGF-1** (ng/ml)	q1	31.2	Ref	**(<0.001)**	26.9	Ref	**(<0.001)**
	q2	41.4	0.274	**0.028**	34.6	0.380	**0.002**
	q3	54.5	0.375	**0.002**	47.9	0.481	<**0.001**
	q4	153	0.771	<**0.001**	223.3	0.831	<**0.001**

^*^Quartile cutoff are denoted as q1 (25th), q2 (50th), q3(75th) and q4 (100th) for individual biomarkers. Q1 was used as a reference quartile for the subsequent comparison of biomarker in each quartile. ^**^Beta estimates of longitudinal LAZ score are shown as change in LAZ per year in a given biomarker quartile. Negative estimates represents the decline in growth with simultaneous increase in biomarkers in a given quartile. P-trend was computed by assigning the median biomarker level in each quartile to participants and evaluating this variable continuously. All P trend values are FDR corrected for Type I error. The list of covariates entered in the model include, sex, preterm birth, maternal age, maternal education status, and use of antibiotics.

At six months of age, the highest quartile of ferritin (>684 ng/ml) was associated with change in LAZ of −0.882 per year during the first 18 months of life, compared to the lowest quartile of ferritin (<13 ng/ml) (p < 0.001). At nine months of age, the highest quartile of ferritin (>370 ng/ml) was associated with change in LAZ of −0.714 per year during the first 18 months of life, compared to the lowest quartile of ferritin (<7 ng/ml) (p < 0.001). The next significant biomarker of systemic inflammation was CRP at 9 months of age. The highest quartile (>103 mg/L) of CRP was associated with change in LAZ of −0.451 per year compared to the reference quartile (<0.3 mg/L) (p < 0.001). The same trend of change in LAZ per year was observed for AGP at 9 month; the highest quartile value of AGP (>312.5 mg/dl) was associated with the rate of change in LAZ of −0.443 per year compared with the reference quartile (84.6 mg/dl) (p < 0.001). To test the hypothesis that suppression of growth hormone is associated with growth faltering in children, we measured IGF-1 hormone in this cohort. As opposed to inflammatory biomarkers, IGF-1 levels were positively associated with change in LAZ per year during first 18 months of life. At 6 month, the children in the highest quartile of IGF-1 (153 ng/ml) had positive increment in LAZ of 0.771 per year compared to the children in the lowest/reference quartile (31.2 ng/ml) (p < 0.001). The same trend was observed at 9 month with the increment of 0.831 LAZ per year (p < 0.001).

Interaction of biomarkers with longitudinal HAZ using continuous biomarkers is also shown in Supplementary Table S2. The rate of change is negligible compared to the analyses of biomarkers in quartiles as shown in Tables 2 and 3.

### Negative association of growth hormone with inflammatory biomarkers

Table 4 shows the correlation analysis of gut and systemic biomarkers with IGF-1 and also shows the interrelationship of these two main groups of biomarkers. At 6 months, the IGF-1 was negatively associated with the majority of gut [GLP2 −0.27 (p < 0.0001), MPO −0.12(p < 0.05), NEO −0.17(p < 0.001)] and systemic inflammatory [Ferritin −0.19 (p < 0.001), CRP −0.28 (p < 0.0001), AGP-0.21 (p < 0.0001)] biomarkers. This relationship remained consistent at 9 month for most of the biomarkers with the exception of Neo and Reg1b. IGF-1 was negatively correlated with AGP (−0.35; p < 0.0001), CRP (−0.30; p < 0.0001) and Ferritin (−0.18; p < 0.001), followed by GLP2 (−0.16; p < 0.001) and MPO (−0.12; p < 0.001). While IGF-1 was strongly correlated inversely with both gut specific and systemic inflammation biomarkers, we found no correlation between the GLP-2 and systemic inflammatory biomarkers, and only a weak correlation between MPO and systemic inflammatory biomarkers at both 6 and 9 months of age (Table 4).

**Table 4:** Correlation of IGF-1 with gut specific and systemic inflammatory biomarkers at 6 and 9 months.

Biomarkers	Ferritin	CRP	AGP	IGF 1	GLP2	MPO	NEO	Reg1b
At 6 months								
Ferritin		0.26***	0.28***	−0.19**	0.105	NS	NS	NS
CRP			0.67***	−0.28***	NS	0.15**	NS	NS
AGP				−0.21***	NS	0.14*	NS	NS
IGF-1					−0.27***	−0.12*	−0.17**	NS
GLP2				−0.27***		NS	0.17**	NS
MPO				−0.12*	NS		NS	
NEO				−0.17**	0.17**	NS		NS
Reg1b				NS	NS	0.23***	NS	
At 9 months								
Ferritin		0.31***	0.38***	−0.18**	0.12*	NS	−0.12*	NS
CRP			0.67***	−0.30***	NS	0.12*	NS	NS
AGP				−0.35***	NS	NS	NS	NS
IGF-1					−0.16**	−0.12*	NS	NS
GLP2				−0.16**		NS	NS	NS
MPO				−0.12*	NS		NS	0.27***
NEO				NS	NS	NS		NS
Reg1b				NS	NS	0.27**	NS	

Note: Values are Spearman’s rank-order correlation coefficient. N = 303 for all 6 month biomarkers and N = 302 for all 9 month biomarkers. ***P < 0.0001; **P < 0.001; *P value < 0.05. Correlation coefficient for negative association between biomarkers are shown in bold and italics, positive association are shown in bold only. NS represents p value > 0.05.

### ROC analysis for the cut off of sensitivity and specificity of biomarkers of EED in children

ROC analysis was performed to determine cutoffs of biomarkers with sensitivity of 80% and corresponding maximum specificity to discriminate between children with change in LAZ during the first 18 months of life. Children with LAZ < −1 were defined as cases and children with LAZ ≥ 0 were considered as reference controls (non-cases) for our ROC analysis as shown in Supplementary Table S3. Among gut specific biomarkers, MPO with c statistics of 0.631 and GLP2 with c statistics of 0.540 were 80% sensitive with specificity ranging between 44% and 22% respectively. Among systemic inflammatory biomarkers, ferritin was found to be discriminatory with c-statistic of 0.747. The cutoffs of >19.55 ng/ml at 6 months and 10.76 ng/ml at 9 months provided 81% sensitivity and corresponding specificity of 59%. The cut-offs of CRP were >0.4 mg/L at 6 months (sensitivity 81%, specificity 32.3%) and >1.0 mg/L at 9 months (sensitivity 80%, specificity 37%).

### Principal component analysis of biomarker at 6 and 9 month

The PCA analysis resolved 6-month biomarkers into four main components (Table 5, Fig. 4). With all eight biomarkers, the PCA showed 60% of variation using 4 major components. The PC1 was associated with biomarkers of inflammation. The highest component loading in PC1 was observed for AGP (0.867), CRP (0.779) and ferritin (0.551), followed by fecal biomarkers, MPO (0.802) and Reg1B (0.591) in PC2. The PC3 and PC4 showed single biomarker as NEO (0.739) and Reg1B serum (0.907) in each of these components. The PCA of 9 month categorized the 9 biomarkers in to three main components. The PC1 include biomarker of inflammation as observed in 6 month, PC2 include IGF-1 (0.588) and NEO (0.707) and Reg1B serum (−0.497) and PC3 include MPO (0.784) and Reg1B fecal (0.736) with highest factor loading.

**Table 5:** Principal component Analysis of biomarkers at 6 and 9 months.

Principal components	6 month biomarkers (n = 301)	Principal components	9 month biomarkers (n = 301)
PC1 (blood)	AGP (0.867), CRP (0.779), Ferritin (0.551) IGF-1 (−0.539)	PC1 (blood)	AGP (0.850), CRP (0.797), Ferritin (0.689)
PC2 (fecal)	MPO (0.802), Reg1(0.591)	PC2 (blood/fecal)	Neo (0.707), IGF-1 (−0.588), Reg1-serum (−0.497)
PC3 (fecal)	Neo (0.739)	PC3 (fecal)	MPO (0.784), Reg1 (0.736)
PC4 (blood)	Reg1B-serum (0.907)		

## Discussion

**Figure 3:**
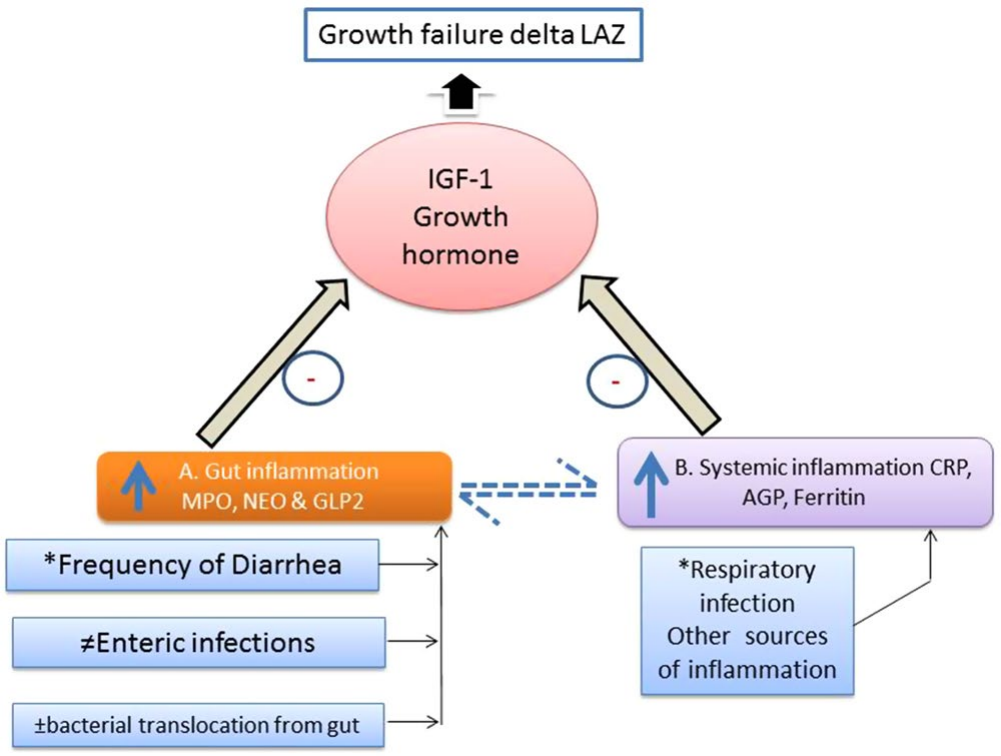
Framework of EED showing gut/systemic inflammation influencing growth in children. This figure describes the hypothetical pathway of EED through the gut (**A**) and systemic inflammation (**B**). Factors driving the inflammatory pathway include respiratory illnesses, enteropathogen infection (measured by TaqMan Array), translocation of bacteria from the gut (IgG/IgA against LPS and Flagellin) due to repeated diarrheal episodes. The two distinct pathways regulating growth hormone, are being measured by gut-specific and systemic biomarkers shown in boxes (**A**) & (**B**). The negative association of biomarkers with IGF results in growth failure in children as shown by the black arrow. The dotted arrows show a weak relationship between the gut and systemic inflammatory biomarkers. *Information collected through weekly morbidity data. ^*≠*^*Estimated through TaqMan array card for bacterial, viral and protozoal targets at 6 and 9 month fecal samples*. ±*IgG/IgA antibodies against LPS and Flagellin was measured in serum samples at 6 and 9 months*.

After establishing a cohort of children at high risk of EED in a rural district of Pakistan, we tested the association of common biomarkers of gut inflammation, regeneration and enterocyte proliferation with growth faltering during the first 18 month of life. We found that two of these biomarkers, GLP-2 and MPO correlated with later growth faltering. Systemic inflammatory markers, including ferritin, CRP and AGP were also negatively associated with linear growth faltering. Both the gut specific and systemic inflammation biomarkers were inversely correlated with the IGF-1 level, which was in turn strongly associated positively with linear growth. We also found that the gut specific biomarkers and the systemic inflammation biomarkers either do not correlate at all, or correlate weakly with each other. We therefore hypothesize that IGF-1 has an independent negative correlation with systemic inflammatory markers, as well as gut specific markers of EED. In other words, the linear growth is mediated by IGF-1, which in turn is influenced by not just systemic inflammation, but also by EED. The influence of EED is not just through its contribution towards systemic inflammation (as shown by weak association between gut specific biomarkers and systemic inflammatory markers), but also its direct influence over IGF-1, perhaps through an independent pathway that does not go through systemic inflammation pathway (as described in the framework of EED in Fig. 3). This hypothesis should be further tested with subsequent EED pathophysiology studies.

**Figure 4:**
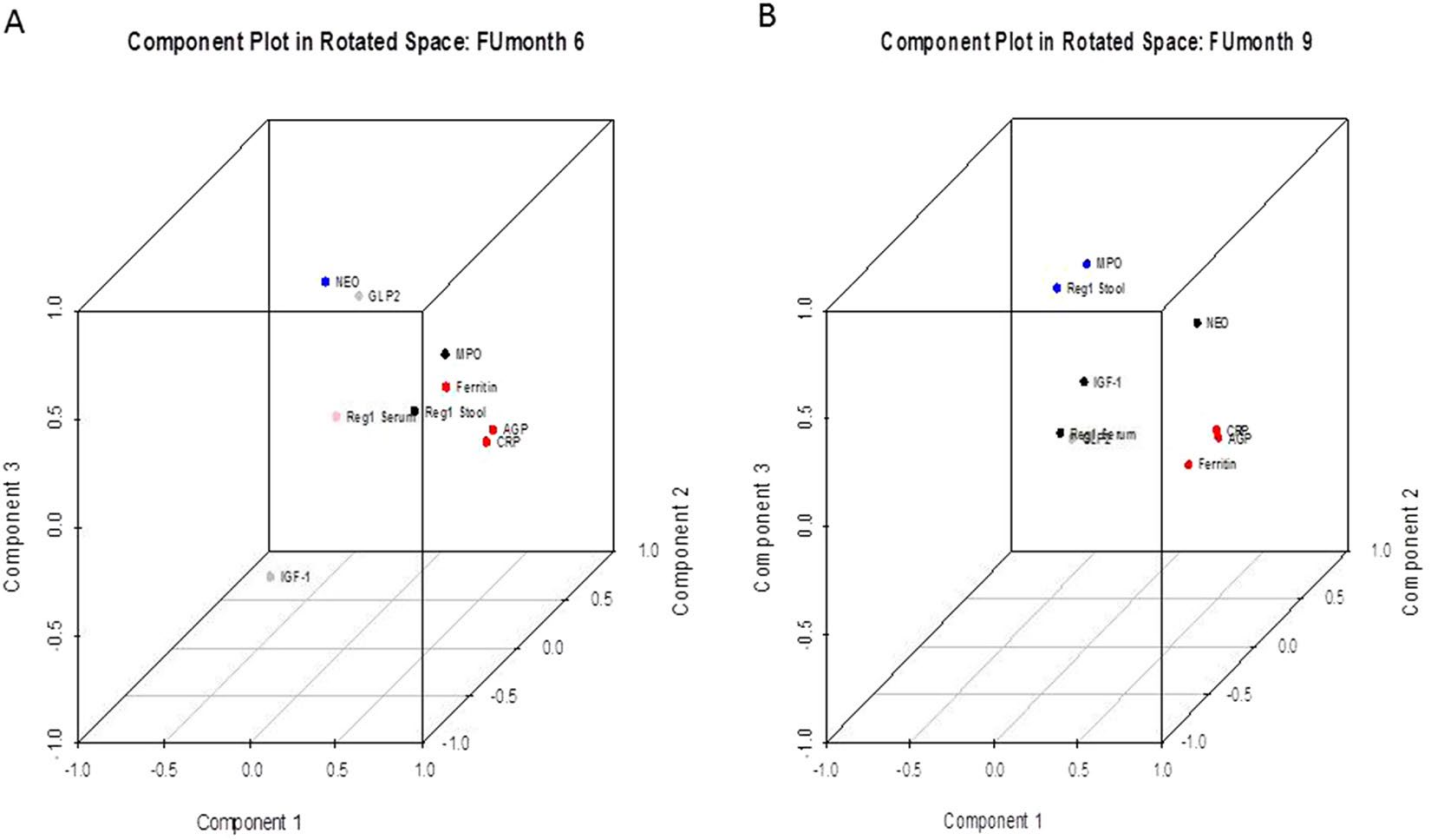
Principal component analysis of EED biomarkers at 6 and 9 months. All biomarkers are shown here in three dimensional plots of 6 and 9 months. Component plot of 6 month biomarker is shown in Fig. 4A. The component loadings of biomarkers of PC1 (systemic inflammation) are shown in red and PC2 (intestinal inflammation) is shown in black. Component plot of 9 month biomarker is shown in Fig. 4B. The component loading of PC1 (Systemic Inflammation) is shown in red. PC2 is shown in black and PC3 is shown in blue as a marker of intestinal inflammation.

In animal model of IBD, similar suppression of growth hormone via pro-inflammatory cytokines^10^ has been described through direct action of these pro-inflammatory mediators on growth plate, or by inhibition of signal transduction by IL6 through Stat 5^11^, by increasing SOCS protein. The low bioavailability of IGFBP3 in protein losing enteropathy and Crohn’s disease partially explained the role of systemic inflammation in decreasing IGF-1 level^12^.

The two gut specific biomarkers, GLP-2 and MPO were correlated with linear growth in our study. GLP2 (3–33) is an intestinotrophic protein, which along with its analogue promote growth and repair of enterocytes in acute inflammatory injury models^13^, and increase nutrient absorption in short bowel syndrome^14^. The trophic effect of GLP2 was also studied in celiac patients before and after gluten free trial, which showed a marked decline in GLP2 suggesting its role in intestinal damage or inflammation^15^. MPO is a marker of neutrophil activity in intestinal mucosa^16^. Both GLP2 and MPO showed negative correlation with IGF-1 hormone, but did not correlate with each other suggesting that both biomarkers are regulated independently, but targeting growth hormone. In the MALED cohort, MPO has been shown to be highly correlated with the presence of enteroinvasive bacteria (campylobacter, shigella, salmonella, ETEC, EPEC and EIEC) in first and second year of life^17^, and negatively associated with delta LAZ and WAZ. We did not find any association of other gut markers like NEO and Reg1. NEO was neither correlated with enteropathogen burden, nor came up in growth modeling, as defined in the MALED cohort. The lack of correlation between these two gut biomarkers in ours and the MALED study explained that both biomarkers represent different pathophysiological pathways and types of immune response^17^.

Reg 1 is a marker of enterocyte proliferation^18,19^ and tissue repair^20–22^, and has been studied in various settings as an EED biomarker. Reg 1α and Reg 1β have 90% homology in protein sequence, and were previously studied in celiac disease^23,24^ and inflammatory bowel disease (IBD)^18,19^. Both of these models are closely related to EED in terms of enterocyte loss and regeneration of crypt cell. A study in Peru and Bangladesh found a strong association of Reg1B level at 3 months with lower LAZ score at 24 month of age^25^. Our lack of association of Reg-1 is interesting given that Peterson and colleague have found some relationship. Of note, in this study, the Reg-1 was measured at 3 months of age while we measured at 6 and 9 months of age. It is plausible that Reg1B may correlate better with growth faltering at younger than 6 month of age.

Our association for systemic inflammatory markers with linear growth is consistent with other studies in the literature. All three systemic inflammatory biomarkers (CRP, AGP, Ferritin) are positively correlated with each other, but negatively correlated with IGF-1^26–28^. Similar associations were observed in Zimbabwean and Kenyan cohort, where AGP, CRP, CD14 and few inflammatory cytokines and chemokines (IL1α, MIP-1β, IFNγ, IL6) were negatively correlated with IGF-1^26,27^.

The PCA analysis of biomarkers is more intuitive in relation to the biological functioning and clustering pattern of these biomarkers. All biomarkers of systemic inflammation clustered together in a distinct cluster as PCI, whereas fecal biomarker, MPO and Reg1 clustered together in PC3 with the exception of NEO. Both NEO and MPO clustered separately, which is also supported by correlation analysis (Table 4). The IGF-1 is interesting as it clustered separately in PC1 in opposite direction for both 6 and 9 month PCA plots. This further corroborates our previous finding of correlation of these inflammatory biomarkers with growth hormone.

Our study has a few limitations. There are various models to assess growth faltering, each having its strengths and limitations. We chose delta LAZ as our primary outcome of interest instead of absolute LAZ at 18 months, in order to account for stunting at the time of birth, which was very common in our cohort. However, our model assumes that post natal growth trajectory of a child stunted at birth would be same as a child not stunted at birth. Given that currently there are no standardized growth charts for children born stunted or normal, we feel justified in working under this assumption. However, this assumption may not be correct, and we know that there are other research groups currently working to develop standardized growth charts for babies born with different anthropometrics (stunted vs. normal etc). Another limitation of our study was a relatively shorter follow up period of 18 months compared to other large cohorts such as the Mal-ED study. This would make measurement of neurocognitive outcomes very difficult in our cohort. Ours is a single center study, and the characteristics of our cohort and study design are somewhat unique (e.g., very high rate of stunting at baseline) compared to other EED cohorts being studied in South East Asian^9,29^ and African countries^26,30^. Biomarker data was not consistently available on all kids, because of the refusal for blood draw on second follow up, drop outs or insufficient volume of blood to carry out analysis for all five biomarkers.

In summary, we found that both systemic inflammation and EED are associated with linear growth, likely through IGF-1. While gut specific biomarkers, GLP-2 and MPO are weak drivers of systemic inflammation, they correlate strongly with IGF-1, which in turn correlates strongly with linear growth. While we are unable to say that GLP-2 and MPO are exclusive correlates of EED, we would like to propose that based on our current study data, these biomarkers should be included in the panel of tests that define EED in future studies that focus on prevention and treatment of this disorder.

## Electronic supplementary material

Supplementary Information
