# Supplementary material for: Promising Biomarkers of Environmental Enteric Dysfunction: A Prospective Cohort study in Pakistani Children
Source: Sci Rep. 2018 Feb 14;8:2966. doi: 10.1038/s41598-018-21319-8 (PMC5813024; doi:10.1038/s41598-018-21319-8)
Supplement: Supplementary file 1 — Supplementary Information [file 41598_2018_21319_MOESM1_ESM.pdf]

Title: Promising Biomarkers of Environmental Enteric Dysfunction: A Prospective Cohort study in Pakistani Children

[1,2]Najeeha Talat Iqbal, [1]Kamran Sadiq, [1,3]Sana Syed, [1]Tauseefullah Akhund, [1]Fayyaz Umrani, [1]Sheraz Ahmed, [1]Mohammad Yawar Yakoob, [1]Najeeb Rahman, [1]Shahida Qureshi, [4]Wenjun Xin, [4]Jennie Z. Ma, [5]Molly Hughes, [1]Syed Asad Ali.

[1]Department of Pediatrics and Child Health, Aga Khan University, Karachi, Pakistan.

[2]Department of Biological and Biomedical Sciences, Aga Khan University, Karachi, Pakistan.

[3] Department of Pediatrics, University of Virginia, Charlottesville, VA, USA.

[4]Department of Public Health Sciences, University of Virginia, USA.

[5]Department of Medicine, University of Virginia, USA.

Table S1. FDR for all 8 Biomarkers in trend test with median value from each quartile

	Biomarker	Raw_pvalue	FDR
6 month	IGF1	0.0000	0.0000
	Ferritin	0.0000	0.0000
	MPO	0.0020	0.0053
	GLP2	0.0179	0.0357
	NEO	0.0612	0.0780
	CRP	0.0680	0.0780
	AGP	0.0683	0.0780
	reg1stool	0.6949	0.6949
9 month	IGF1	0.0000	0.0000
	Ferritin	0.0000	0.0000
	AGP	0.0045	0.0119
	CRP	0.0197	0.0394
	GLP2	0.0283	0.0453
	NEO	0.0412	0.0549
	MPO	0.2647	0.3025
	reg1stool	0.6460	0.6460

Table S2. A. Association of slope of HAZ with continuous estimation of biomarker of systemic inflammation at 6 and 9 month

Biomarker	6 month		9 month	
	Year*Biomarker (slope of LAZ)	pvalue	Year*Biomarker (slope of LAZ)	pvalue
Ferritin (ng/ml)	-0.00405	**<0.0001**	-0.00607	**<0.0001**
CRP (mg/L)	-0.14416	**0.0156**	-0.11414	**0.0051**
AGP (mg/dL)	-0.00239	**0.0299**	-0.00346	**0.0005**
IGF-1 (ng/ml)	0.00997	**<0.0001**	0.01219	**<0.0001**

Table S2. B. Association of slope of HAZ with continuous estimation of biomarker of intestinal inflammation at 6 and 9 month

Biomarker	6 month		9 month	
	Year*Biomarker (slope of LAZ)	pvalue	Year*Biomarker (slope of LAZ)	pvalue
GLP (pg/ml)	1.19819E-06	0.4928	-7.00217E-06	0.1543
MPO (ng/ml)	-5.202E-06	**0.029**	-5.47886E-06	0.1014
NEO (nmol/L)	-2.40887E-05	0.7743	3.59536E-06	0.9650
Reg1b (pg/ml)	-4.52741E-05	0.8316	0.000111421	0.5426

Beta estimates of longitudinal LAZ score are shown as change in LAZ per year with continuous biomarker. Negative estimates represent the decline in growth with simultaneous change in overall biomarker profile. Data was controlled for covariates such as sex, pre-term birth, maternal age, maternal education and antibiotic use.

Table S3 **ROC analysis of biomarkers at 6 and 9 months in children with HAZ<-1.0 and HAZ>0 from birth to 18th month**

**Biomarkers**	**6 month**			**9 month**		
	**Cutoff**	**Sensitivity**	**Specificity**	**Cutoff**	**Sensitivity**	**Specificity**
Ferritin (ng/ml)	>19.55	81.3	58.5	>10.76	81.8	45.2
CRP (mg/L)	>0.4	81.3	32.3	>1.0	80	37.1
AGP (mg/dL)	>66.28	80.8	34.5	>83.17	80.5	39
IGF-1 (ng/ml)	<53.90	80.8	34.5	<45.14	80	47
GLP (pg/ml)	>12097	80.7	22	>4435	80	22
MPO (ng/ml)	>4810	80.8	23.9	>2611	80	44
NEO (nmol/L)	>733	82.3	19.5	>304	80	18
Reg1b (pg/ml)	>346	80.6	20.3	>288	81	18

Suppl figure 1 : Time line of follow up of cohort from April 2013-May 2015

Months →	0	1	2	3	4	5	6	7	8	9	10	11	12	13	14	15	16	17	18
Anthropometric assessment	x	x	x	x	x	x	x	x	x	x	x	x	x	x	x	x	x	x	x
Blood							x			x									
Stool							x			x									
Urine							x			x									
Nutritional Intervention																			
Educational Intervention																			
